# Doxycycline post-exposure prophylaxis for sexually transmitted infections in South Africa

**DOI:** 10.4102/sajhivmed.v24i1.1510

**Published:** 2023-09-28

**Authors:** Remco P.H. Peters, James A. McIntyre, Nigel Garrett, Adrian J. Brink, Connie L. Celum, Linda-Gail Bekker

**Affiliations:** 1 Research Unit, Foundation for Professional Development, East London, South Africa; 2 Department of Medical Microbiology, University of Pretoria, Pretoria, South Africa; 3 Division of Medical Microbiology, University of Cape Town, Cape Town, South Africa; 4 Anova Health Institute, Johannesburg, South Africa; 5 School of Public Health and Family Medicine, University of Cape Town, Cape Town, South Africa; 6 Centre for the AIDS Programme of Research in South Africa, University of KwaZulu-Natal, Durban, South Africa; 7 Department of Public Health Medicine, School of Nursing and Public Health, University of KwaZulu-Natal, Durban, South Africa; 8 Division of Medical Microbiology, University of Cape Town, Cape Town, South Africa; 9 National Health Laboratory Services, Groote Schuur Hospital, Cape Town, South Africa; 10 Institute of Infectious Disease and Molecular Medicine, University of Cape Town, Cape Town, South Africa; 11 Departments of Global Health, Medicine, and Epidemiology, University of Washington, Seattle, United States of America; 12 Desmond Tutu HIV Centre, University of Cape Town, Cape Town, South Africa

**Keywords:** doxycycline, STI prevention, men who have sex with men, antimicrobial resistance, *Chlamydia trachomatis*, *Neisseria gonorrhoeae*, syphilis

## Abstract

South Africa has a large burden of bacterial sexually transmitted infections (STIs) with high rates among men who have sex with men (MSM). Randomised controlled trials have recently demonstrated high effectiveness of doxycycline post-exposure prophylaxis (PEP) for prevention of bacterial STIs in MSM, with 70% – 85% reductions in *Chlamydia trachomatis* infection and syphilis, and approximately 50% reduction in *Neisseria gonorrhoeae* infection. Doxycycline PEP was not demonstrated to be effective in reducing *C. trachomatis* and *N. gonorrhoeae* infection among Kenyan cisgender women. Although no worrisome trends in antimicrobial resistance (AMR) were observed in the trials, important concerns remain about doxycycline PEP and AMR development in STIs, other pathogens, commensals, and the microbiome. Tetracycline resistance in *N. gonorrhoeae* is already widespread in South Africa, but emergence of AMR in other STIs would be concerning. Larger sample sizes of doxycycline PEP users with longer follow-up time are needed to understand the impact that doxycycline PEP may have on AMR at individual and population level. In this opinion article, we weigh the benefits of doxycycline PEP for prevention of bacterial STIs against the existing AMR concerns and data gaps in the South African context. Based on the current evidence, we conclude that it would be reasonable to offer doxycycline PEP to high-risk MSM on a case-by-case basis, provided that it is offered by experienced sexual health clinicians in settings that have access to diagnostic STI testing and ongoing AMR surveillance.

**What this study adds:** This opinion article discusses doxycycline post-exposure prophylaxis (PEP) in the context of the South African healthcare system. Benefits and concerns of doxycycline PEP are discussed, and recommendation for implementation in controlled settings is provided.

## Sexually transmitted infection burden in South Africa

South Africa has one of the largest burdens of bacterial sexually transmitted infections (STIs) worldwide. Estimates of the World Health Organization (WHO) Spectrum STI model are that each year 3.9 million new cases of *Chlamydia trachomatis*, 2.2m new cases of *Neisseria gonorrhoeae* and 47 500 new cases of active syphilis infection occur in men in South Africa.^1^ In women, the estimated number of annual new cases is 1.9m of *C. trachomatis*, 2.3m of *N. gonorrhoeae*, and 23 175 of active syphilis.^1^ Men who have sex with men (MSM) are disproportionally affected. Prevalence estimates for *C. trachomatis* (12% – 26%), *N. gonorrhoeae* (8.1% – 28%) and active syphilis (5.2% – 17%) reported in research studies of MSM exceed that of the general male population (6.0%, 3.5% and 0.97%, respectively) (Table 1); *Mycoplasma genitalium* prevalence of 8.1% was reported in MSM.^1,2,3,4,5,6^ Incidence data are limited but one study reported a combined incidence of *C. trachomatis* and *N. gonorrhoeae* of 188/100 person-years in MSM in Johannesburg.^5^

**TABLE 1:** Prevalence and incidence estimates of curable sexually transmitted infections in men who have sex with men in South Africa.

Author (year)	Study details	Prevalence (%)					Incidence (per 100 PY)		
		CT	NG	MG	Syphilis	CT	NG	MG (UR)	Syphilis
Le Roux et al. (2023)	Cross-sectional study of STI prevalence in 200 MSM in Tshwane	18	17	8.1	-	-	-	-	-
Mashingaidze et al. (2023)	Cohort analysis of HIV-uninfected MSM participating in the HVTN 702 phase 2b/3 trial of HIV preventive vaccine	26	8.1	-	5.2	-	-	-	-
Jones et al. (2020)	Prospective cohort study of 263 MSM and 22 TGW in Cape Town and Port Elizabeth to inform HIV prevention package								
	Urethral	10	3.0		17	13	7.1	-	8.2
	Rectal	16	25		-	33	278	-	-
Van Liere et al. (2019)	Prospective cohort study of 78 MSM presenting with urethral or rectal discharge in Johannesburg								
	Urethral	-	-	-	-	17	85	26	-
	Rectal	-	-	-	-	26	60	-	-
Rebe et al. (2015)	Cross-sectional study of 200 MSM attending a sexual health services clinic in Cape Town	12	16	-	11	-	-	-	-

Source: Please see the full reference list of the article Le Roux M, Ngwanya IK, Numarude AL. Sexually transmitted infections and sexual behaviour among men have sex with men from Tswhane, South Africa. Int J STD AIDS. 2023;34(3):183–190. https://doi.org/10.1177/09564624221146673, for more information

CT, *Chlamydia trachomatis*; NG, *Neisseria gonorrhoeae*; MG, *Mycoplasma genitalium*; STI, sexually transmitted infection; MSM, men who have sex with men; TGW, transgender women; PY, person-years.

Interventions are urgently needed to reduce the burden of STIs, associated morbidity and potential impact on HIV transmission. Recently, several randomised controlled trials (RCT) have reported on doxycycline prophylaxis as an effective, well-tolerated, and acceptable novel biomedical approach to prevent bacterial STIs among MSM. We discuss the effectiveness, concerns about antimicrobial resistance (AMR), and considerations for implementation of doxycycline prophylaxis for bacterial STIs in South Africa.

## Doxycycline for sexually transmitted infections

Doxycycline is a bacteriostatic antibiotic, a second-generation tetracycline with broad antibacterial spectrum that acts on the ribosomal protein synthesis unit.^7^ Its liposolubility ensures high bioavailability and high tissue and fluid penetration. For STI post-exposure prophylaxis (PEP), a single dose of 200 mg doxycycline was used, ideally to be taken within 24 h (maximum 72 h) after condomless sex.^8,9^

Doxycycline (100 mg twice daily) is used for syphilis treatment of non-pregnant adults in the South African syndromic regimen for genital ulcer disease due to global benzathine benzylpenicillin shortage.^10^

Although azithromycin covers *C. trachomatis* and *M. genitalium* in the syndromic regimen for genital discharge,^10^ in case of diagnostic test, the Southern African HIV Clinicians Society’s guidelines recommend doxycycline for first-line pathogen-directed treatment of *C. trachomatis*, and as part of the sequential therapy regimen for documented symptomatic *M. genitalium* infection.^11^ Doxycycline is not recommended for *N. gonorrhoeae* due to the high levels of antimicrobial resistance (AMR), with up to 90% of strains being resistant to tetracyclines.^12,13,14^

Other than STIs, doxycycline is used at the primary healthcare level for treatment of acne vulgaris, tick bite fever, in patients with an acute exacerbation of chronic obstructive pulmonary disease who have severe penicillin allergy, and as malaria prophylaxis.^15^

## Effectiveness of doxycycline prophylaxis for bacterial sexually transmitted infections

Three RCTs have demonstrated the efficacy of doxycycline PEP for STI prevention in MSM and one in women (Table 2).^16,17,18,19^ The IPERGAY trial from France reported a 47% reduction in STI incidence in the doxycycline PEP group of MSM taking HIV pre-exposure prophylaxis (PrEP).^16^ The relative reduction was almost 70% for *C. trachomatis* and syphilis, but there was no reduction in *N. gonorrhoeae*. The DOXYVAC trial by the same investigators observed similar reductions in STI in MSM taking HIV PrEP and with a history of STI treatment with an 84% reduction in *C. trachomatis* and syphilis and 51% reduction in *N. gonorrhoeae*.^17^

**TABLE 2:** Key characteristics of randomised controlled trials of doxycycline prophylaxis for bacterial sexually transmitted infections.

Study	Country	Study details	Study population	Findings							
				Any STI		NG		CT		Syphilis	
				HR† or RR‡	95% CI	HR† or RR‡	95% CI	HR† or RR‡	95% CI	HR† or RR‡	95% CI
Sub-study of ANRS IPERGAY Molina et al. (2018)	France	**Design:** Open-label RCT of doxyPEP versus standard of care (1:1) **Primary endpoint:** occurrence of first STI during 10 months follow-up	232 Men who have condomless sex with men and are using HIV PrEP	0.53†	0.33–0.85	0.83†	0.47–1.47	0.30†	0.13–0.70	0.27†	0.07–0.98
ANRS 174 DOXYVAC Molina et al. (2023)	France	**Design:** 2 × 2 RCT of doxyPEP vs. standard of care (2:1) and 4CMenB vaccine vs. no vaccine (1:1) **Primary endpoint:** time to first episode of CT or syphilis and time to first NG episode	502 asymptomatic MSM on HIV PrEP > 6 months, enrolled in ANRS Prevenir, and with bacterial STI in prior 12 months	0.16†	0.08–0.30§	0.49†	0.32–0.76	0.11†	0.04–0.40	0.21†	0.09–0.47
DoxyPEP Luetkemeyer et al. (2023)	US	**Design:** Open-label RCT of doxyPEP vs. standard of care (2:1) **Primary endpoint:** incidence of at least one STI per follow-up quarter	432 MSM or TGW taking HIV PrEP who had had an STI† in the past year	0.34‡	0.24–0.46	0.45‡	0.32–0.65	0.12‡	0.05–0.25	0.13‡	0.03–0.59
		**Design:** RCT of doxyPEP vs. standard of care (2:1) **Primary endpoint:** incidence of at least one STI per follow-up quarter	209 MSM or TGW living with HIV who had had an STI† in the past year	0.38‡	0.24–0.60	0.43‡	0.26–0.71	0.26‡	0.12–0.57	0.23‡	0.04–1.29
dPEP Kenya Stewart et al. (2023)	Kenya	**Design:** RCT of doxyPEP vs. standard of care (1:1) **Primary endpoint:** any incident STI measured quarterly for one year	449 cisgender women (18–30 years) taking HIV PrEP	0.88‡	0.60–1.29	1.64‡	0.78–3.47	0.73‡	0.47–1.13	-	-

Source: Please see the full reference list of the article Molina JM, Charreau I, Chidiac C, et al. Post-exposure prophylaxis with doxycycline to prevent sexually transmitted infections in men who have sex with men: An open-label randomised substudy of the ANRS IPERGAY trial. Lancet Infect Dis. 2018;18(3):308–317. https://doi.org/10.1016/S1473-3099(17)30725-9, for more information

ANRS, agence nationale de recherche sur le sida et les hépatites virales; HR, hazard ratio; RR, relative risk; CI, confidence interval; STI, sexually transmitted infection; NG, *Neisseria gonorrhoeae*; CT, *Chlamydia trachomatis*; RCT, randomised controlled trial; PrEP, pre-exposure prophylaxis; MSM, men who have sex with men; TGW, transgender women.

† , HR data;

‡ , RR data;

§ , For any CT or syphilis.

The DoxyPEP trial in the United States (US) of MSM and transgender women (TGW) with a history of STIs in the prior year also showed reductions in STI incidence among those using HIV PrEP (66% overall) and in people living with HIV (PLHIV) (62% overall).^18^ Participants received three bottles of 30 doxycycline tablets and were counselled to take 200 mg of doxycycline, ideally within 24 h but no later than 72 h after condomless anogenital, vaginal, or oral sex, and not more than one dose every 24 h. In persons taking HIV PrEP, doxycycline PEP reduced syphilis by 87% and chlamydia by 88% while reductions of 77% in syphilis and 74% in chlamydia were observed for PLHIV. Unlike the IPERGAY trial, this study showed a reduction of *N. gonorrhoeae* incidence of 55% in PrEP users, and 57% among PLHIV. Some of the variability in the efficacy of doxycycline PEP against *N. gonorrhoeae* in the two French trials compared to the DoxyPEP trial in the US could be explained by the lower background level of doxycycline resistance in the US compared to France at the time of the trials, and that DoxyPEP trial participants were permitted to have more doses than in IPERGAY.^20^

Unlike the efficacy observed in the three trials among MSM, the dPEP Kenya trial did not show efficacy of doxycycline PEP in cisgender women (18–30 years).^19^ A nonsignificant decrease in *C. trachomatis* was observed, but the number of *N. gonorrhoeae* cases increased in the doxycycline PEP arm. Furthermore, only one case of syphilis occurred during the study period, making it impossible to draw conclusions on the effectiveness against syphilis. Various explanations for the lack of efficacy in these young women are under investigation; initial results indicate adherence is a major factor with approximately half of 50 randomly selected participants having a hair sample with detectable doxycycline.^21^

## Doxycycline prophylaxis and antimicrobial resistance

The main concern about doxycycline PEP is the effect it may have on AMR, other bacterial pathogens, and on the human microbiome.^20^ The IPERGAY and DoxyPEP trials did not observe a statistically significant increase in doxycycline resistance in *N. gonorrhoeae* strains although the sample sizes were small and with relatively short follow-up (median of 9 months).^16,18^ Similarly, oropharyngeal commensal *Neisseria* species, which may constitute a risk for AMR through horizontal gene transfer, did not significantly increase between baseline (63%) and follow-up (70%) in the doxycycline PEP arm in the DoxyPEP trial.^22^ However, the impact of doxycycline PEP on AMR in *N. gonorrhoeae* may be limited in South Africa as studies report that 74% – 89% of *N. gonorrhoeae* strains are already resistant to tetracyclines.^12,13,14^ Also, hypothetical concerns about co-selection of resistance to other classes of antibiotics may be limited given that most *N. gonorrhoeae* strains already have the putative *tet*(M) and *rpsJ* V57M mutations.^13,14,23^

There is little evidence of AMR in *C. trachomatis* and *T. pallidum* despite widespread use of doxycycline for these infections.^9,20^ Rare reports of treatment failure have been published.^20^ Resistance could theoretically emerge through single-point mutations or gene transfer, but this is generally considered unlikely.^16,18,20,22^ *M. genitalium* has the potential to acquire tetracycline resistance given that this organism has a history of rapidly acquiring resistance mutations. Despite the low rate (< 2%) of resistance to azithromycin,^24,25^ previously as stand-alone treatment, sequential therapy of doxycycline followed by azithromycin or moxifloxacin is now recommended to avoid further emergence of AMR.^11^ The effects of doxycycline PEP on development of AMR in *M. genitalium* are unknown but warrant close monitoring.

Treatment with tetracyclines has been associated with AMR in ‘bystander’ respiratory and gastrointestinal tract pathogens.^20^ Data from a limited number of prospective studies suggest that treatment with oral tetracyclines for 2–18 weeks may increase resistance in the bacterial flora, although the effects may be modest and transient.^26^ The effects of doxycycline PEP on the microbiome may be different than when used daily for treatment; for example, the median number of pills taken in the DOXYVAC trial was 7 per month (interquartile range [IQR]: 4–11) and 4 per month (IQR: 1–10) in the DoxyPEP trial.^17,18^ Nevertheless, the DoxyPEP trial reported a decrease from 42% to 29% in nasopharyngeal carriage of *S. aureus* in a small sample of doxycycline PEP users, with an increase from 4% to 12% in the proportion of doxycycline-resistant strains between baseline and 1 year follow-up.^22^ The DoxyPEP study reported a persistently high (> 80%) prevalence of nasopharyngeal commensal *Neisseria* species in the doxycycline PEP arm, also with a nonsignificant increase in proportion of doxycycline-resistant isolates (63% to 70%).^22^ Studies with larger sample sizes and longer follow-up time are required to better understand impact of doxycycline PEP on STIs, other pathogens and the microbiome. This could be done by close monitoring and AMR surveillance of indiviudals using doxycycline PEP in health programmes.

## Balancing effectiveness of doxycycline post-exposure prophylaxis and concerns of antimicrobial resistance

Doxycycline is a well-known, well-tolerated antibiotic that has been used for decades in the treatment of infectious diseases.^7^ Randomised controlled trials have shown efficacy of doxycycline PEP for STI prevention in MSM, but not in young cisgender women, while efficacy has not been evaluated in female sex workers and men who are not MSM.^16,17,18^ Therefore, consideration of doxycycline PEP for STI prevention should be limited to MSM at this stage.

Incidence of STIs in MSM in South Africa are higher than reported in the trials, suggesting that the number needed to treat to prevent one STI may be smaller than 4.7 in MSM on HIV PrEP and 5.3 in PLHIV, as reported in the DoxyPEP trial.^18^ The preventive effect would be predominantly to reduce *C. trachomatis* and *T. pallidum* infections due to the high rate of tetracycline resistance in *N. gonorrhoeae*.^12,13,14^ Given the high burden, associated morbidity and effects on HIV transmission, the efficacy of doxycycline PEP, and the limited alternative preventive options available, it would be reasonable to offer doxycycline PEP for primary STI prevention as part of a comprehensive sexual health package to high-risk MSM.

Diagnostic STI testing should be performed before prescribing doxycycline PEP to avoid suboptimal treatment and potentially driving AMR of any undiagnosed prevalent infection. Resources permitting, diagnostic testing should include sensitive nucleic acid amplification tests for oropharyngeal, urethral, and anorectal *C. trachomatis* and *N. gonorrhoeae* infection as well as syphilis serology.^11^ Diagnostic tests for STIs are not routinely available in South Africa, but there are settings where access to diagnostic tests can be organised, for example in the private sector, research studies and implementation demonstration projects. Importantly, access to diagnostic STI testing and biomedical preventions for MSM are key objectives of the WHO’s Global Health Sector strategies on HIV, viral hepatitis and STIs,^27^ as well as the National Strategic Plan for HIV, tuberculosis and STIs 2023–2028.^28^

There are many unanswered questions and genuine concerns about the long-term effects of doxycycline PEP on AMR in STIs, other pathogens and the microbiome.^20^ The impact of doxycycline PEP on AMR in *N. gonorrhoeae* may be relatively small given that most strains are resistant to tetracyclines.^12,13,14^ Emergence of doxycycline resistance in other STIs, especially *M. genitalium*, would be highly concerning given the limited treatment options available. Therefore, we recommend that doxycycline PEP is offered in well-controlled settings and with access to diagnostic testing (including AMR) by clinicians with expertise in STI management.

The potential effects of doxycyline PEP on AMR in other pathogens and the microbiome should be considered in the South African context. Doxycycline has only a few indications, alternative treatment options are generally available and AMR development is unlikely (e.g., tick bite fever and malaria prophylaxis).^13^ Furthermore, tetracyclines are used widespread as the primary antibiotic in agriculture;^29^ this may have more impact on driving population-level AMR than the effects of doxycycline PEP use by a small subgroup. On the other hand, intensive use of doxycycline in a small core group of users may select or induce AMR in pathogens that could subsequently spread to the general population.^18^ Although no worrisome trends in AMR were observed in the doxycycline PEP trials, further research is warranted to better understand the effects of doxycycline PEP on tetracycline resistance at both the individual and population level.

Globally, the balance between effectiveness of doxycycline PEP and AMR concerns is weighed differently. The San Francisco Public Health Unit, the California Department of Public Health and the Public Health Seattle & King Country in the US recommend doxycycline PEP use by MSM and TGW with a recent history of STI.^30,31,32^ In the absence of long-term data regarding the impact of doxycycline PEP on AMR and the microbiome, the Centers for Disease Control and Prevention (CDC) provides considerations to healthcare providers to inform decision-making on a case-by-case basis, and additional guidelines are anticipated later in 2023.^33^ The Australasian Society for HIV, Viral Hepatitis and Sexual Health Medicine is planning to convene a forum in 2023 to provide evidence-based guidance and recommendations.^9^ The British Association for Sexual Health and HIV and the United Kingdom (UK) Health Security Agency do not endorse doxycycline for STI prophylaxis for lack of data about AMR, while other countries are waiting for further data before making formal recommendations.^34^ The WHO has not yet issued any statement.

Doxycycline PrEP (200 mg daily) is being evaluated as an alternative to PEP. One Canadian RCT comparing doxycycline PrEP and doxycycline PEP has started and an Australian study is giving MSM the option of doxycycline PEP or PrEP. These studies follow a pilot RCT that showed a 73% reduction in bacterial STIs in a sample of 30 MSM.^35^ However, at this stage, there is insufficient evidence to recommend daily doxycycline PrEP.

## Conclusion

Doxycyline PEP is a novel and effective preventive intervention that could substantially reduce the high STI burden and associated morbidity in MSM in South Africa. These STI prevention benefits should be weighed against the largely unknown risks and potential future harms of AMR. In the absence of current local doxycycline PEP data, it is essential that South Africa builds expertise in doxycycline PEP provision and improves STI and AMR surveillance. Based on current evidence, we think it is reasonable to offer doxycycline PEP to high-risk MSM on a case-by-case basis following comprehensive sexual health counselling by experienced clinicians in settings that have access to diagnostic STI testing and ongoing AMR surveillance.

## References

[1] Kularatne RS, Nitt R, Rowley J, et al. Adult gonorrhea, chlamydia and syphilis prevalence, incidence, treatment, and syndromic case reporting in South Africa: Estimates using the Spectrum-STI model, 1990–2017. PLoS One. 2018;13(10):e0205863. 10.1371/journal.pone.0205863 30321236 PMC6188893

[2] Le Roux M, Ngwanya IK, Numarude AL. Sexually transmitted infections and sexual behaviour among men have sex with men from Tswhane, South Africa. Int J STD AIDS. 2023;34(3):183–190. 10.1177/09564624221146673 36542494

[3] Mashingaidze R, Moodie Z, Allen M, et al. Sexually transmitted infections amongst men who have sex with men (MSM) in South Africa. PLoS Glob Public Health. 2023;3(4):e0001782. 10.1371/journal.pgph.0001782 37018240 PMC10075439

[4] Jones J, Sanchez TH, Dominguez K, et al. Sexually transmitted infection screening, prevalence and incidence among South African men and transgender women who have sex with men enrolled in a combination HIV prevention cohort study: The Sibanye Methods for Prevention Packages Programme (MP3) project. J Int AIDS Soc. 2020;23(suppl. 6):e25594. 10.1002/jia2.25594 33000886 PMC7527766

[5] Van Liere GAFS, Kock MM, Radebe O, et al. High rate of repeat sexually transmitted diseases among men who have sex with men in South Africa: A prospective cohort study. Sex Transm Dis. 2019;46(11):e105–e107. 10.1097/OLQ.0000000000001041 31268955

[6] Rebe K, Lewis D, Myer L, et al. A cross-sectional analysis of gonococcal and chlamydial infections among men-who-have-sex-with-men in Cape Town, South Africa. PLoS One. 2015;10(9):e0138315. 10.1371/journal.pone.0138315 26418464 PMC4587970

[7] Peyriere H, Makinson A, Marchandin H, Reynes J. Doxycycline in the management of sexually transmitted infections. J Antimicrob Chemother. 2018;73(3):553–563. 10.1093/jac/dkx420 29182717

[8] Grant JS, Stafylis C, Celum C, et al. Doxycycline prophylaxis for bacterial sexually transmitted infections. Clin Infect Dis. 2020;70(6):1247–1253. 10.1093/cid/ciz866 31504345 PMC7319058

[9] Cornelisse VJ, Ong JJ, Ryder N, et al. Interim position statement on doxycycline post-exposure prophylaxis (Doxy-PEP) for the prevention of bacterial sexually transmissible infections in Australia and Aotearoa New Zealand – The Australasian Society for HIV, Viral Hepatitis and Sexual Health Medicine (ASHM). Sex Health. 2023;20(2):99–104. 10.1071/SH23011 36927481

[10] National Department of Health. Sexually transmitted infections management guidelines [homepage on the Internet]. 2018, p. 5–8. Retrieved 16 August 2023. Available from: https://www.health.gov.za/wp-content/uploads/2020/11/sti-guidelines-27-08-19.pdf

[11] Peters RPH, Garrett N, Chandiwana N, et al. Southern African HIV clinicians Society 2022 guideline for the management of sexually transmitted infections: moving towards best practice. S Afr J HIV Med. 2022;23(1):1450. 10.4102/sajhivmed.v23i1.1450 PMC9575338 36299557

[12] Kularatne R, Maseko V, Gumede L, Kufa T. Trends in *Neisseria gonorrhoeae* antimicrobial resistance over a ten-year surveillance period, Johannesburg, South Africa, 2008–2017. Antibiotics. 2018;7(3):58. 10.3390/antibiotics7030058 30002329 PMC6165174

[13] Maduna LD, Kock MM, Van der Veer BMJW, et al. Antimicrobial resistance of *Neisseria gonorrhoeae* isolates from high-risk men in Johannesburg, South Africa. Antimicrob Agents Chemother. 2020;64(11):e00906-20. 10.1128/AAC.00906-20 32868325 PMC7577157

[14] Mitchev N, Singh R, Allam M, et al. Antimicrobial resistance mechanisms, multilocus sequence typing, and NG-STAR sequence types of diverse *Neisseria gonorrhoeae* isolates in KwaZulu-Natal, South Africa. Antimicrob Agents Chemother. 2021;65(10):e0075921. 10.1128/AAC.00759-21 34280016 PMC8448096

[15] The National Department of Health, South Africa: Essential Drugs Programme. Primary healthcare standard treatment guideline and essential medicine list. 7th ed. Pretoria: South African National Department of Health; 2020.

[16] Molina JM, Charreau I, Chidiac C, et al. Post-exposure prophylaxis with doxycycline to prevent sexually transmitted infections in men who have sex with men: An open-label randomised substudy of the ANRS IPERGAY trial. Lancet Infect Dis. 2018;18(3):308–317. 10.1016/S1473-3099(17)30725-9 29229440

[17] Molina JM, Bercot B, Assoumou L, et al. ANRS 174 DOXYVAC: An open-label randomized trial to prevent STIs in MSM on PrEP. Conference on Retroviruses and Opportunistic Infections (CROI), 19–22 February 2023, Seattle, WA; 2023.

[18] Luetkemeyer AF, Donnell D, Dombrowski JC, et al. Postexposure doxycycline to prevent bacterial sexually transmitted infections. N Engl J Med. 2023;388(14):1296–1306. 10.1056/NEJMoa2211934 37018493 PMC10140182

[19] Stewart J, Oware K, Donnell D, et al. Doxycycline postexposure prophylaxis for prevention of STIs among cisgender women. Conference on Retroviruses and Opportunistic Infections (CROI), 19–22 February 2023, Seattle, WA; 2023.

[20] Kong FYS, Kenyon C, Unemo M. Important considerations regarding the widespread use of doxycycline chemoprophylaxis against sexually transmitted infections. J Antimicrob Chemother. 2023;78(7):1561–1568. 10.1093/jac/dkad129 37129293 PMC10577522

[21] Stewart J, Oware, Donnell D, et al. Adherence to doxycycline PEP for STI prevention among cisgender women. ISSTDR and IUSTI STI & HIV World Congress, 24–27 July 2023, Chicago, IL.

[22] Luetkemeyer AF, Donnell D, Dombrowski JC, et al. Doxy-PEP and antimicrobial resistance in *S. aureus*, *N. gonorrhoeae*, and commensal Neisseria. Conference on Retroviruses and Opportunistic Infections (CROI), 19–22 February 2023, Seattle, WA; 2023.

[23] Vanbaelen T, Manoharan-Basil SS, Kenyon C. Doxycycline post exposure prophylaxis could induce cross-resistance to other classes of antimicrobials in *Neisseria gonorrhoeae*: An in-silico analysis. Sex Transm Dis. 2023;50(8):490–493. 10.1097/OLQ0000000000001810 36952471

[24] Mahlangu MP, Muller EE, Da Costa Dias B, Venter JME, Kularatne RS. Molecular characterization and detection of macrolide and fluoroquinolone resistance determinants in Mycoplasma genitalium, South Africa, 2015 to 2018. Sex Transm Dis. 2022;49(7):511–516. 10.1097/OLQ.0000000000001631 35312667

[25] Peters RPH, Jung HS, Muller EE, et al. Lack of macrolide resistance in *Mycoplasma genitalium* infections in a cohort of pregnant women in South Africa. Sex Transm Infect. 2021;97(8):624–625. 10.1136/sextrans-2020-054583 33542152 PMC9168781

[26] Truong R, Tang V, Grennan T, Tan DHS. A systematic review of the impacts of oral tetracycline class antibiotics on antimicrobial resistance in normal human flora. JAC Antimicrob Resist. 2023;4(1):dlac009. 10.1093/jacamr/dlac009 PMC8855662 35198979

[27] World Health Organization. Global health sector strategies on, respectively, HIV, viral hepatitis and sexually transmitted infections for the period 2022–2030. Geneva: World Health Organization; 2022.

[28] South African National AIDS Council. National Strategic Plan for HIV, TB, and STIs 2023–2028 [homepage on the Internet]. Note date. [cited 2023 Aug 16]. Available from: https://sanac.org.za/wp-content/uploads/2023/05/SANAC-NSP-2023-2028-Web-Version.pdf

[29] Mupfunya CR, Qekwana DN, Naidoo V. Antimicrobial use practices and resistance in indicator bacteria in communal cattle in the Mnisi community, Mpumalanga, South Africa. Vet Med Sci. 2021;7(1):112–121. 32865348 10.1002/vms3.334 PMC7840202

[30] San Francisco Department of Public Health. Health update: Doxycycline post-exposure prophylaxis reduces incidence of sexually transmitted infections [homepage on the Internet]. October 2022. Available from: https://www.sfcdcp.org/wp-content/uploads/2022/10/Health-Update-Doxycycline-Post-Exposure-Prophylaxis-Reduces-Incidence-of-Sexually-Transmitted-Infections-SFDPH-FINAL-10.20.2022.pdf

[31] California Department of Public Health. Doxycycline post-exposure prophylaxis (doxy-PEP) for the prevention of bacterial sexually transmitted infections (STIs) [homepage on the Internet]. Available from: https://www.cdph.ca.gov/Programs/CID/DCDC/CDPH%20Document%20Library/CDPH-Doxy-PEP-Recommendations-for-Prevention-of-STIs.pdf

[32] Public Health Seattle & King Country. Doxycycline post-exposure prophylaxis (Doxy-PEP) to prevent bacterial STIs in men who have sex with men (MSM) and transgender persons who have sex with men. Available from: https://kingcounty.gov/depts/health/communicable-diseases/~/media/depts/health/communicable-diseases/documents/hivstd/DoxyPEP-Guidelines.ashx

[33] CDC. Primary prevention methods. Postexposure prophylaxis for HIV and STIs [homepage on the Internet]. Available from: https://www.cdc.gov/std/treatment-guidelines/clinical-primary.htm#CautionsForDoxyPEP

[34] Kohli M, Medland N, Fifer H, Saunders J. BASHH updated position statement on doxycycline as prophylaxis for sexually transmitted infections. Sex Transm Infect. 2022;98(3):235–236. 10.1136/sextrans-2022-055425 35414633 PMC9016249

[35] Bolan RK, Beymer MR, Weiss RE, et al. Doxycycline prophylaxis to reduce incident syphilis among HIV-infected men who have sex with men who continue to engage in high-risk sex: A randomized, controlled pilot study. Sex Transm Dis. 2015;42(2):98–103. 10.1097/OLQ0000000000000216 25585069 PMC4295649

